# Supplementary material for: Prolonged normothermic perfusion of the kidney prior to transplantation: a historically controlled, phase 1 cohort study
Source: Nat Commun. 2025 May 17;16:4584. doi: 10.1038/s41467-025-59829-5 (PMC12085653; doi:10.1038/s41467-025-59829-5)
Supplement: Supplementary file 1 — Supplementary Information [file 41467_2025_59829_MOESM1_ESM.pdf]

NKP1 – Supplementary Information

Statistics and graphical representation

Throughout this Supplementary Information, the following points apply:

- Points and error bars (figures S4A, S5G-I, S9I, S10B,C) represent means ± SD.
- Boxplots (figures S4B, S6B-D, S9 A,B,D,F-H S10A,D-F,H,I): centre lines shown are medians; bounds of the boxes are first and third quartiles; whiskers extend to either the most extreme value or the limit of the box ± 1.5x IQR, whichever is closest to the median; individual datapoints are overlaid and jittered.
- Continuously recorded machine data (figures S5A,B,D-F) are shown with a solid line representing the group mean, and the shaded area indicating the mean ± SD.

Figure S1: Control cohort flow diagram

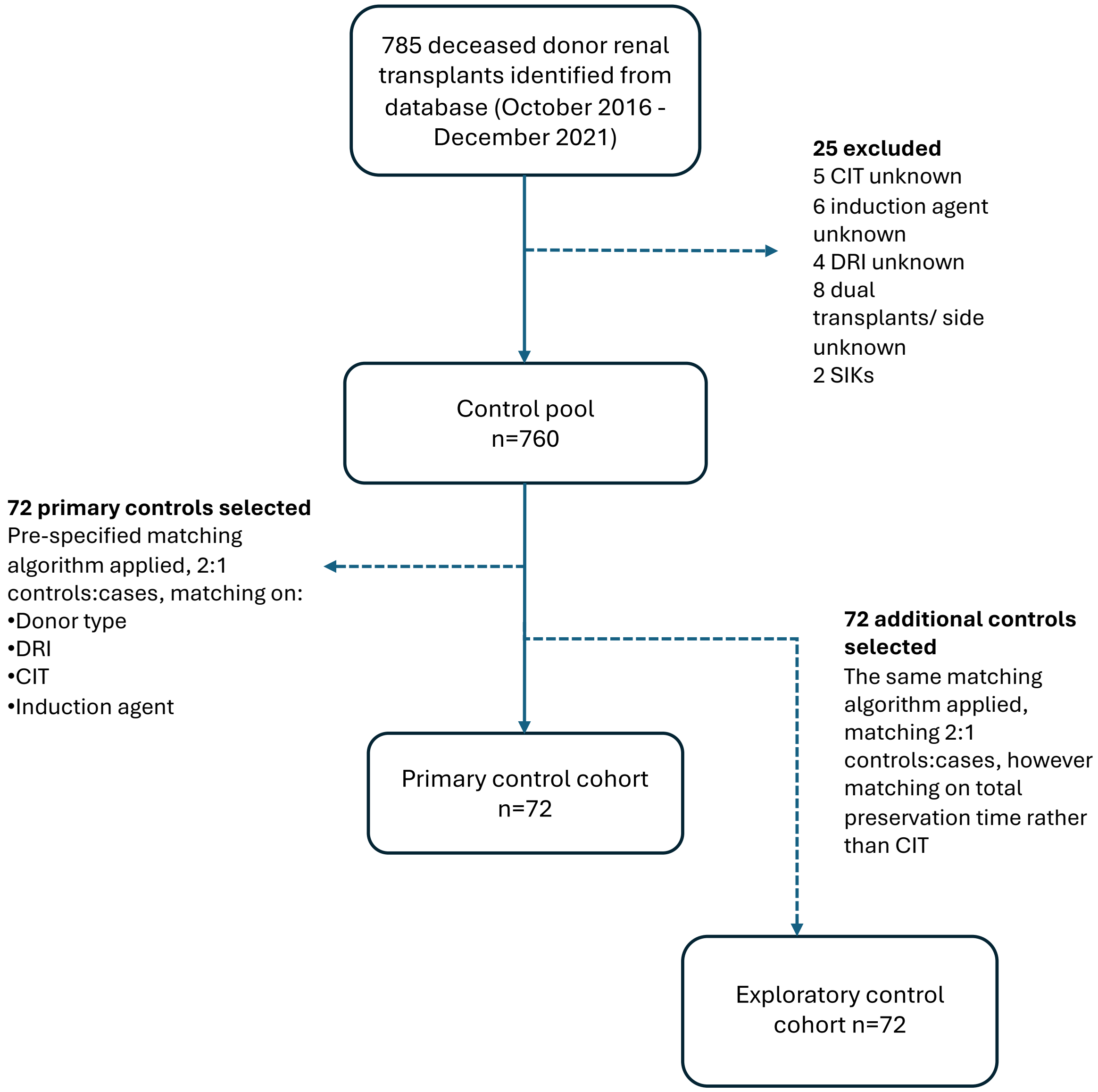

Figure S1: Flow diagram showing selection of the control cohort. CIT: cold ischaemic time. DRI: donor risk index. SIK: simultaneous islet-kidney transplant.

Table S2: Serious adverse events table

	NMP ≤6 hours (N = 20)	NMP 6-12 hours (N = 12)	NMP 12-24 hours (N = 4)	Total (N = 36)
**Number of participants who experienced a serious adverse event during the one-year follow-up period, n (%)**	8 (40.0)	7 (58.3)	3 (75.0)	18 (50.0)
Infection	3 (15.0)	1 (8.3)	3 (75.0)	7 (19.4)
Biopsy-proven acute rejection	1 (5.0)	0 (0.0)	0 (0.0)	1 (2.8)
Ureteric complication	1 (5.0)	0 (0.0)	0 (0.0)	1 (2.8)
Vascular complication	1 (5.0)	1 (8.3)	0 (0.0)	2 (5.6)
Other	5 (25.0)	6 (50.0)	0 (0.0)	11 (30.6)
**Number of serious adverse events during the one-year follow-up period, n**	17	12	5	34
Infection	9	1	5	10
Biopsy-proven acute rejection	1	0	0	1
Ureteric complication	1	0	0	1
Vascular complication	1	1	0	2
Other	5	10	0	15
**Number of deaths, n (%)**	1 (8.3)	0 (0.0)	0 (0.0)	1 (2.8%)
**Number of graft failures, n (%)**	1 (8.3)	0 (0.0)	0 (0.0)	1 (2.8%)

Table S2: Table detailing the serious adverse events recorded during the follow-up period (first 12 months following transplantation) in the trial cohort. Serious adverse events listed as 'other' included musculoskeletal (subacute vertebral compression fracture, n=1; pubic ramus fracture, n=1; gout, n=1); gastro-intestinal (diarrhoea associated with AKI, n=2; GI ulceration/ inflammation/ bleeding, n=2); cardiovascular (heart block, n=1); bleeding related to surgery (n=2); seroma (n=1); pyrexia of unknown origin (n=1); incisional hernia (n=2); and graft failure (n=1). Note that the number of participants with SAEs in each sub-category does not necessarily sum to the total number of participants with an SAE, as participants may have experienced an SAE in more than one sub-category.

Table S3: NMP microbiology

Organism	Hypothermic transport perfusion fluid (number of cultures)	NMP perfusate (number of cultures)	Notes
E. coli	2	0	
Klebsiella oxytoca	1	0	
Enterococcus faecium	1	1	Same case; enterococcus faecium carried through from hypothermic to normothermic perfusate. Recipient given prophylactic course of linezolid; no clinical evidence of related infection.
Candida albicans	1	0	
Staphylococcus epidermidis	3	0	
Coagulase negative staphylococcus (species not specified)	4	0	
Lactobacillus paracasaei	1	0	
Lactobacillus rhamnosus	1	1	Same case; lactobacillus rhamnosus carried through from hypothermic to normothermic perfusate. Recipient not treated, and no clinical evidence of infection.
Weissella cibaria	1	0	

Table S3: Table detailing organisms grown in perfusate for the 36 trial cases – data are shown for both the hypothermic preservation fluid that the organ was transported to Oxford in, and the normothermic perfusion fluid used during the intervention.

Figure S4: Clinical outcomes in cases and controls

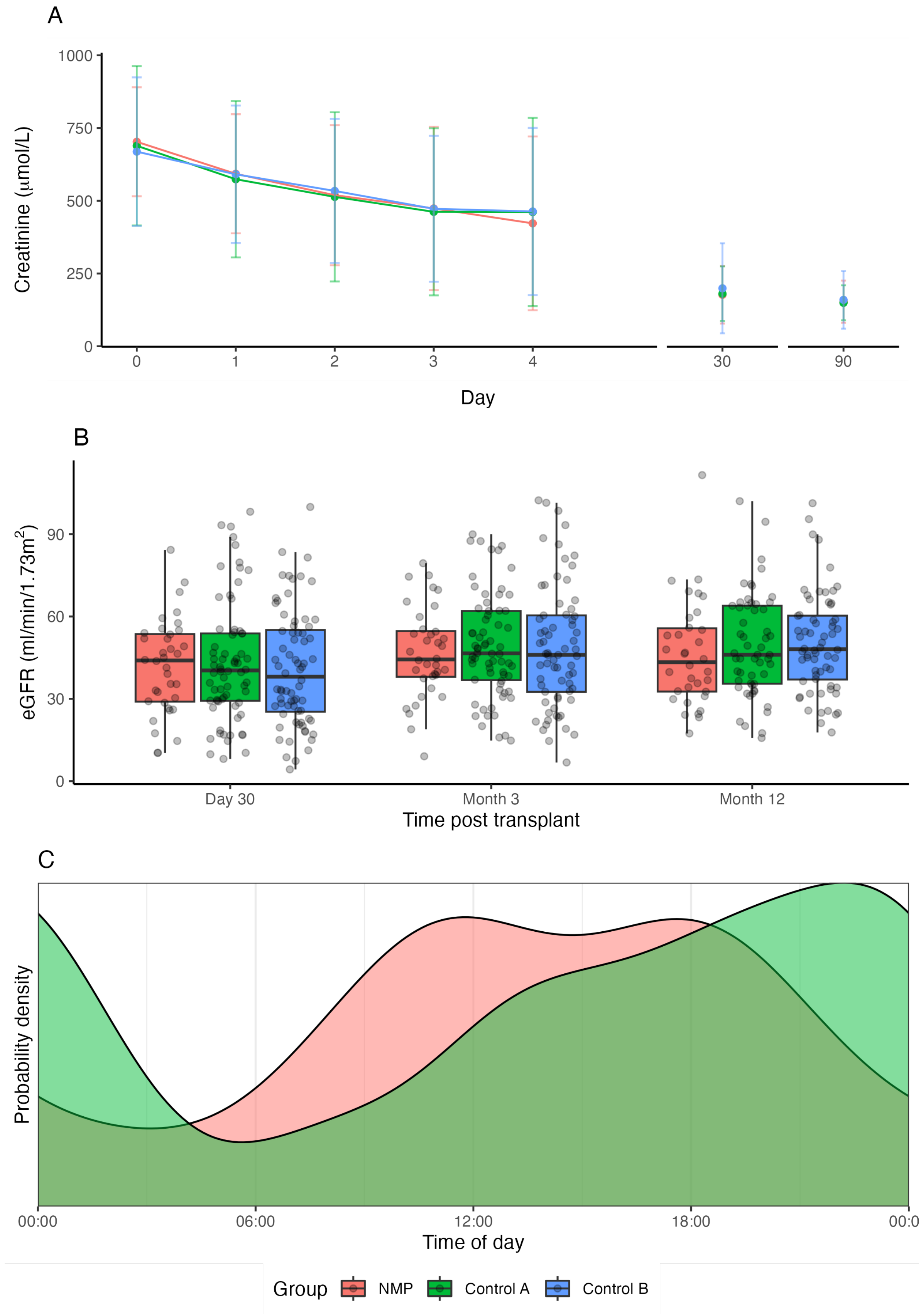

Figure S4: Clinical outcomes seen post transplantation in the cases and controls. Control cohort A was the primary control cohort matched on cold ischaemia time; control cohort B was an additional exploratory cohort matched on total preservation time. A: creatinine (mean±SD) in the first four days post-transplant, at 30 days, and at 90 days. B: calculated eGFR in all three cohorts at 30 days, 3 months, and 12 months post-transplant. C: probability density plot showing time of day of reperfusion in the trial cases, and the primary control cohort. Source data are provided as a Source Data File.

Figure S5: Additional perfusion biochemistry and machine data

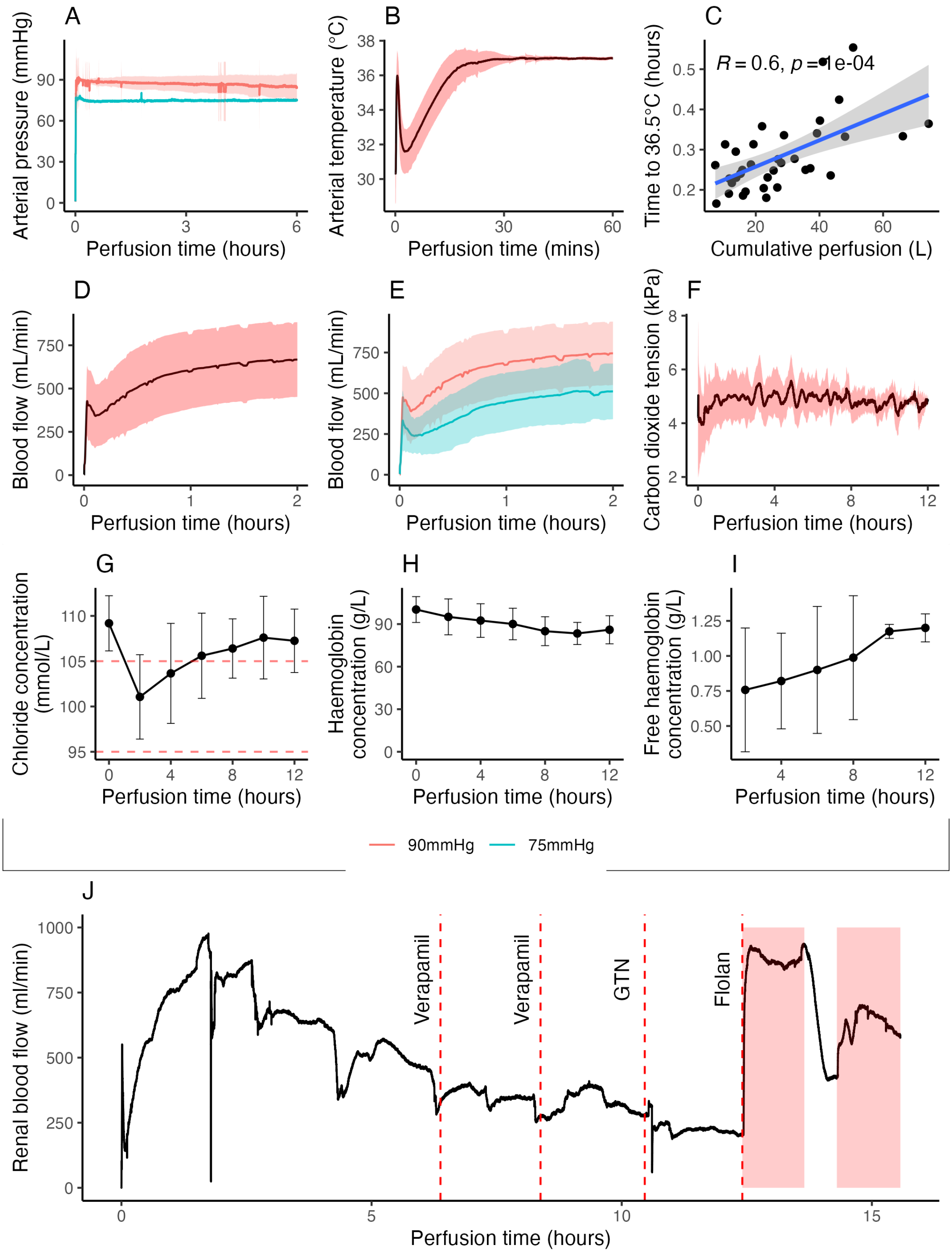

Figure S5: Additional perfusate biochemistry and machine data. A: Pressure during perfusion, split by cohort (first 24 cases perfused at 90mmHg, last 12 cases perfused at 75mmHg). B: perfusate temperature following ex-situ reperfusion (n=36, mean±SD). C: time taken to re-warm to 36.5°C, against cumulative perfusion volume to that time (linear regression, Pearson's correlation coefficient shown, n=36, shaded area indicates 95% CI). D: renal blood flow during the first two hours of perfusion (n=36, mean±SD). E: renal blood flow split by perfusion pressure group (n=24 at 90mmHg, n=12 at 75mmHg, mean±SD). F: carbon dioxide tension in the perfusate during perfusion (mean±SD). G: perfusate chloride concentration during perfusion (mean±SD, dashed red lines indicate normal range 95-105mmol/L). H: perfusate total haemoglobin concentration during perfusion (mean±SD). I: perfusate cell-free haemoglobin concentration during perfusion (mean±SD). A, and F-I: for number of kidneys perfused to each time point see Figure 3 legend. J: renal blood flow over time for a single case (kidney 30), illustrating effect of different vasodilators. Source data are provided as a Source Data File.

Figure S6: NMP dose effect (perfusion duration vs outcome)

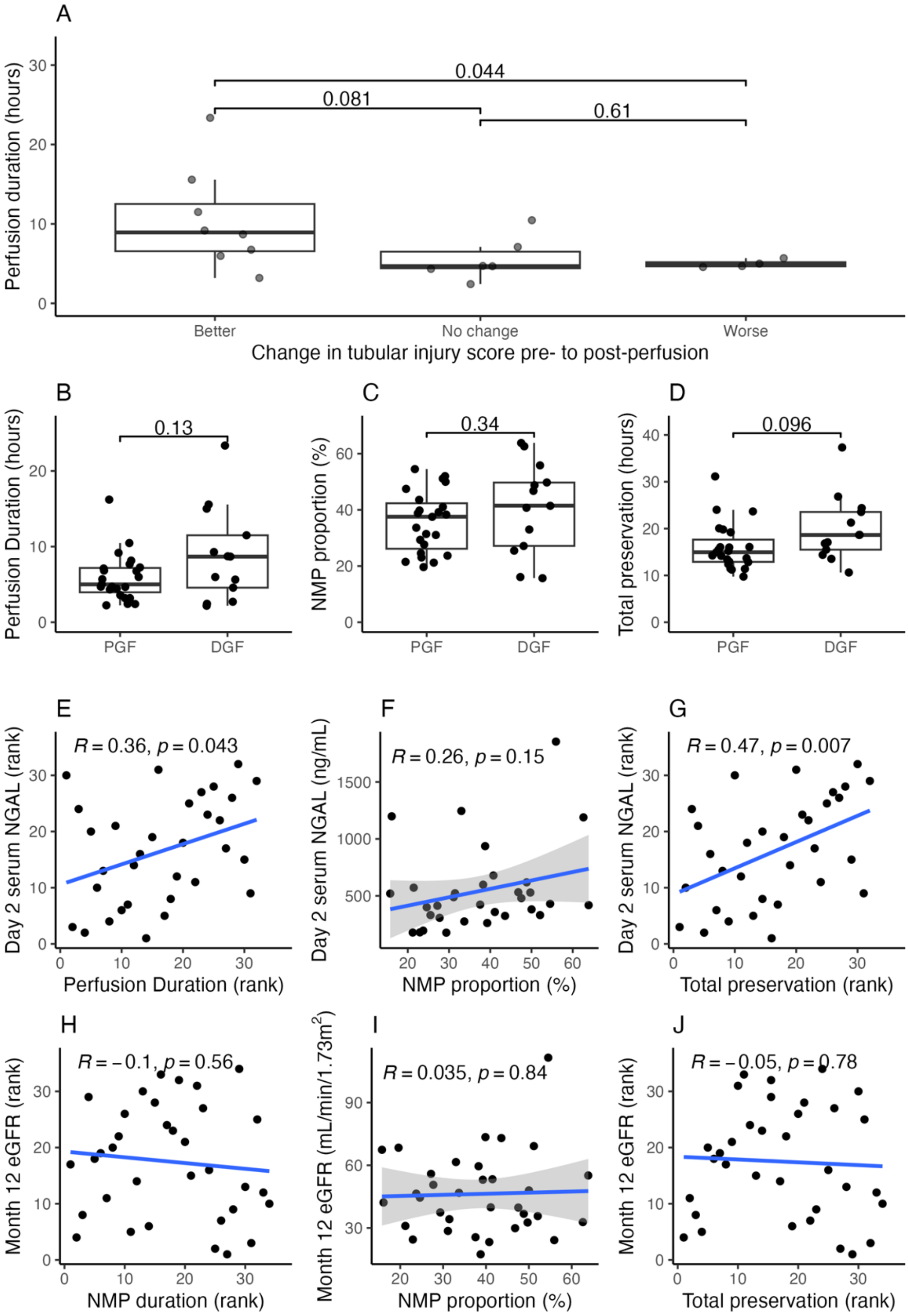

Figure S6: Evaluation of the effect of NMP duration on outcome. A: pre- and post-perfusion biopsies were assessed by an independent pathologist blinded to perfusion duration. Acute tubular injury scores were compared pre- and post-perfusion, and classified as better post-perfusion, unchanged, or worse post-perfusion (Welch's T-test, n=18). B-D: the median perfusion duration (B, Welch's T-test, two-sided), mean NMP proportion of the total preservation time (C, T-test, two-sided), and median total preservation time (D, Welch's T-test, two-sided) was compared between immediate function and DGF groups (n=36). E-G: post-transplant day 2 NGAL concentration correlated against perfusion duration (E), NMP proportion of the total preservation time (F), and perfusion duration (G, all by linear regression, Pearson correlation coefficient shown for the proportion, Spearman correlation coefficient and ranked data shown for the durations, n=32). H-J: month 12 eGFR was correlated against perfusion duration (H), NMP proportion of the total preservation time (I), and perfusion duration (J, all by linear regression, Pearson correlation coefficient shown for the proportion, Spearman correlation coefficient and ranked data shown for the durations, n=34). H and I: shaded areas indicate 95% confidence intervals. Source data are provided as a Source Data File.

Table S7: Additional control cohort demographic data

Variable		NKP1	Additional control cohort
**Timings**			
First CIT, hours, median (IQR)		8.57 (3.49)	15.33 (6.36)
Normothermic machine perfusion time, hours, median (IQR)		5.83 (4.54)	-
Second CIT, minutes, median (IQR)		68.4 (30)	-
Total preservation time, hours, median (IQR)		15.74 (6.35)	15.33 (6.36)
**Recipients**			
Age, years, mean (sd)		59.2 (12.0)	56.1 (13.0)
Sex, male, n (%)		24/36 (67%)	49/72 (68%)
BMI, mean (sd)		28.1 (5.8)	29.4 (6.3)
Dialysis modality, n (%)	HD	22/36 (61%)	45 (63%)
	PD	10/36 (28%)	15 (21%)
	Predialysis	4/36 (11%)	12 (17%)
Induction immunosuppression, n (%)	Alemtuzumab	26/36 (72%)	52/72 (72%)
	Basiliximab	10/36 (28%)	20/72 (28%)
**Donors**			
Donor age, years, mean (sd)		54.0 (15.8)	52.4 (15.1)
Donor type, n (%)	DBD	20/36 (56%)	40 (56%)
	DCD	16/36 (44%)	32 (44%)
Donor sex, male, n (%)		23/36 (64%)	42/72 (58%)
Donor BMI, mean (sd)		28.3 (6.4)	27.2 (5.5)
Donor history of hypertension, yes, n (%)		18/36 (50%)	29/72 (40%)
Donor risk index (DRI), mean (sd)		1.36 (0.59)	1.35 (0.56)
HLAMM group, n (%)*	1	0/36 (0%)	6/68 (9%)
	2	9/36 (25%)	10/68 15%)
	3	19/36 (53%)	34/68 (50%)
	4	8/36 (22%)	18/68 (26%)

Table S7: Demographic data for the addition, exploratory, control cohort matched on total preservation time. CIT: cold ischaemic time. HD: haemodialysis. PD: peritoneal dialysis. IQR: interquartile range. HLAMM: human leucocyte antigen mismatches. DRI: donor risk index. Source data are provided as a Source Data File.

Table S8: Additional control cohort outcomes

Variable	NKP1	Additional control cohort	Comparison
30-day graft survival , n (%)	36/36 (100%)	72/72 (100%)	NS
30-day patient survival, n (%)	36/36 (100%)	72/72 (100%)	NS
3-month patient survival, n (%)	36/36 (100%)	70/72 (97%)	NS
12-month patient survival, n (%)	35/36 (97%)	70/72 (97%)	NS
3-month graft survival, n (%)	36/36 (100%)	70/70 (100%)	NS
12-month graft survival, n (%)	34/35 (97%)	69/70 (99%)	NS
DGF (dialysis, any) - incidence, n (%)	13/36 (36%)	25/72 (35%)	NS
fDGF - incidence, n (%)	18/36 (50%)	41/69 (59.4%)	NS
Day 2 CRR, mean (SD)	0.16 (0.20)	0.09 (0.22)	NS
PNF, n (%)	0/36 (0%)	0/72 (0%)	NS
30-day eGFR, mean (SD)	41.9 (17.4)	40.9 (21.0)	NS
3-month eGFR, mean (SD)	46.5 (16.4)	48.7 (22.4)	NS
12-month eGFR, mean (SD)	46.3 (19.3)	50.3 (18.2)	NS
Creatinine gradient (month 3-12), mean (SD)	-2 (46)	-8 (38)	NS
Biopsy-proven acute rejection within 12 months - incidence, n (%)	1/36 (2.8%)	5/72 (6.9%)	NS

Table S8: Clinical outcomes for the addition, exploratory, control cohort matched on total preservation time. DGF: delayed graft function. HD: haemodialysis. fDGF: functional delayed graft function (any use of dialysis in the first 7 days post-transplant or failure of creatinine to fall >10% per day for the first three days). CRR: creatinine reduction ratio. PNF: primary non-function. eGFR: estimated glomerular filtration rate. Source data are provided as a Source Data File.

Figure S9: Additional biomarker data (function)

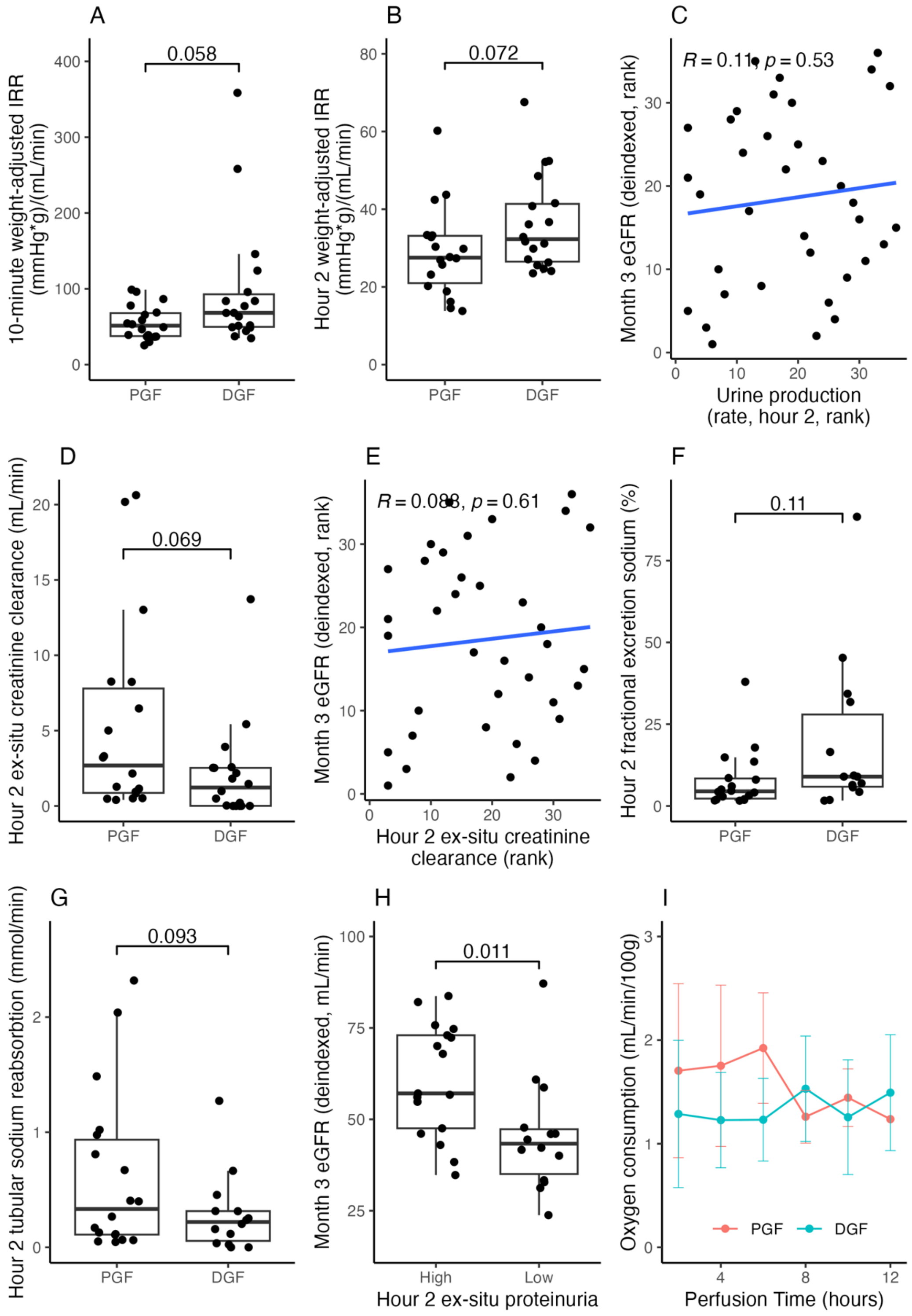

Figure S9: Additional biomarkers (markers of ex-situ renal function). A and B: weight-adjusted vascular resistance measured at minute 10 (A) and hour 2 (B), compared between functional DGF and immediate-function cohorts (Welch's T-tests, n=36, two-sided). C: cumulative urine output to hour 2 correlated against deindexed month 3 eGFR (linear regression on ranked data, Spearman correlation coefficient shown, n=36). D and E: ex-situ creatinine clearance, measured at hour 2, shown against immediate function status (D, Welch's T-test, n=36, two-sided) and month 3 eGFR (E, linear regression on ranked data, Spearman correlation coefficient shown, n=36). F and G: fractional excretion of sodium (F), and the tubular sodium absorption rate (G), both measured at hour 2, split by immediate function status (Welch's T-tests, n=32, two-sided). H: proteinuria, quantified at hour 2 and dichotomised at ≥4g/L versus <4g/L, shown against deindexed month 3 eGFR (T-test, n=31, two-sided). I: oxygen consumption over time during perfusion, split by immediate function status (for number of kidneys perfused to each time point see Figure 3 legend). Source data are provided as a Source Data File.

Figure S10: Additional biomarker data (injury)

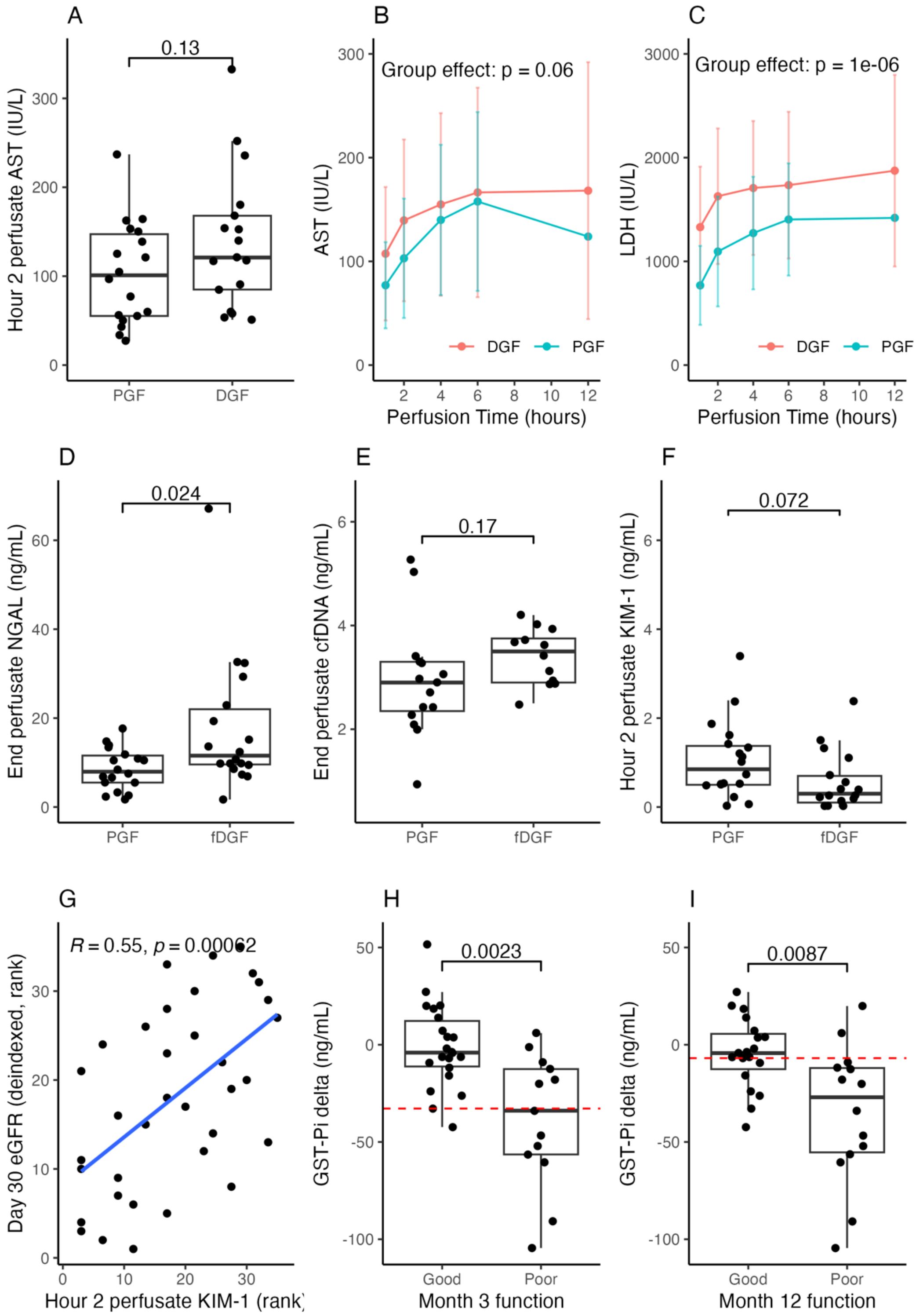

Figure S10: Additional biomarkers (measures of ex-situ renal injury). A and B: perfusate AST, split by immediate function status, measured at hour 2 (A, T-test, n=35, two-sided), and over time during perfusion (B, two-way analysis of variance). C: LDH over time during perfusion (two-way analysis of variance). B and C: for number of kidneys perfused to each time point see Figure 3 legend. D-F: NGAL (D, n=36), cell-free DNA (E, n=27), and KIM-1 (F, n=35) measured in the perfusate at the end of perfusion, split by immediate function status (cfDNA – T-tests, NGAL and KIM-1 – Welch's T-tests, all two-sided). G: perfusate KIM-1 (end of perfusion) correlated against day 30 eGFR (linear regression on ranked data, Spearman correlation coefficient shown, n=35). H and I: the trial cohort was divided into good and poor functional groups using an eGFR cut-off of 45mL/min. Delta GST-Pi (difference between perfusate concentration at hour 2 and at the end of perfusion) was compared between these groups at 3- and 12-months post-transplant (T-tests, two-sided). Red dashed lines indicated classifier cut-offs identified by ROC analysis (H: n=35; I: n=33). Source data are provided as a Source Data File.
