# Supplementary material for: Prolonged normothermic perfusion of the kidney prior to transplantation: a historically controlled, phase 1 cohort study
Source: Nat Commun. 2025 May 17;16:4584. doi: 10.1038/s41467-025-59829-5 (PMC12085653; doi:10.1038/s41467-025-59829-5)
Supplement: Supplementary file 2 — Reporting Summary [file 41467_2025_59829_MOESM2_ESM.pdf]

nature portfolio

Corresponding author(s): Richard Dumbill
Constantin Coussios

Last updated by author(s): Mar 9, 2025

Reporting Summary

Nature Portfolio wishes to improve the reproducibility of the work that we publish. This form provides structure for consistency and transparency in reporting. For further information on Nature Portfolio policies, see our Editorial Policies and the Editorial Policy Checklist.

Statistics

For all statistical analyses, confirm that the following items are present in the figure legend, table legend, main text, or Methods section.

n/a	Confirmed	
☐	☒	The exact sample size (*n*) for each experimental group/condition, given as a discrete number and unit of measurement
☐	☒	A statement on whether measurements were taken from distinct samples or whether the same sample was measured repeatedly
☐	☒	The statistical test(s) used AND whether they are one- or two-sided<br>*Only common tests should be described solely by name; describe more complex techniques in the Methods section.*
☐	☒	A description of all covariates tested
☐	☒	A description of any assumptions or corrections, such as tests of normality and adjustment for multiple comparisons
☐	☒	A full description of the statistical parameters including central tendency (e.g. means) or other basic estimates (e.g. regression coefficient) AND variation (e.g. standard deviation) or associated estimates of uncertainty (e.g. confidence intervals)
☐	☒	For null hypothesis testing, the test statistic (e.g. *F*, *t*, *r*) with confidence intervals, effect sizes, degrees of freedom and *P* value noted<br>*Give P values as exact values whenever suitable.*
☒	☐	For Bayesian analysis, information on the choice of priors and Markov chain Monte Carlo settings
☒	☐	For hierarchical and complex designs, identification of the appropriate level for tests and full reporting of outcomes
☐	☒	Estimates of effect sizes (e.g. Cohen's *d*, Pearson's *r*), indicating how they were calculated

Our web collection on statistics for biologists contains articles on many of the points above.

Software and code

Policy information about availability of computer code

Data collection	Study data were collected and managed using REDCap electronic data capture tools, hosted at Oxford University.
Data analysis	Data analysis was conducted in R Studio, Version 2023.06.0+421

For manuscripts utilizing custom algorithms or software that are central to the research but not yet described in published literature, software must be made available to editors and reviewers. We strongly encourage code deposition in a community repository (e.g. GitHub). See the Nature Portfolio guidelines for submitting code & software for further information.

Data

Policy information about availability of data

All manuscripts must include a data availability statement. This statement should provide the following information, where applicable:

- Accession codes, unique identifiers, or web links for publicly available datasets
- A description of any restrictions on data availability
- For clinical datasets or third party data, please ensure that the statement adheres to our policy

NKP1 was a single centre phase 1 trial with a limited number of participants. In order to protect individual patient confidentiality, a limited trial dataset containing the source data required to reproduce the key figures is provided with this paper. The protocol is also publicly available (https://doi.org/10.1186/ISRCTN13292277). Reasonable written requests addressed to the corresponding authors, for access to the full trial dataset (individual de-identified participant and perfusion data), will be considered on a case-by-case basis within two months of receipt. Adequate protection of participant confidentiality will be the primary factor governing further data sharing.

Research involving human participants, their data, or biological material

Policy information about studies with human participants or human data. See also policy information about sex, gender (identity/presentation), and sexual orientation and race, ethnicity and racism.

Reporting on sex and gender	NKP1 was open to donor organs and transplant recipients of all sexes and genders, and the recruited cohort reflects this (see Table 1). Sex and gender were not explicitly considered by the study design, and so a priori sex- or gender-based analyses have not been performed. Sex was determined as that assigned at birth. Results are not presented disaggregated by sex or gender due to the low numbers inherent in a phase 1 trial, however some individual-level data are provided in the Source Data file.
Reporting on race, ethnicity, or other socially relevant groupings	Neither donor or recipient race, ethnicity, nor other socially relevant groupings are explicitly reported in this manuscript. Ethnicity data is indirectly used in that it is a component of the CKD-EPI (2009) formula for determining estimated glomerular filtration rate, which is an important post-transplant variable. CKD-EPI (2009) was the current version at the time the protocol was written and was explicitly specified in advance as the method for calculation of post-transplant eGFR. For this calculation ethnicity as a binary variable (black or non-black) is required. During NKP1, self-report was used for this categorisation.
Population characteristics	Age, sex, organ donor type (DBD or DCD), BMI, recipient dialysis modality and duration, donor and recipient medical histories as reported in demographic table 1.
Recruitment	Recipients on the waiting list for kidney transplantation were consulted whilst on the waiting list (in advance) about the trial (a provision of information step, rather than formal consent). This provision of information step consisted of both written study information (the participant information sheet) and a telephone consultation with an investigator. Where required, translation services were used. In all cases participants had a least 24 hours to consider this information, and in most cases substantially longer. Participants who had indicated during this provision of information consultation that they were interested in joining the trial were approached if they were called in for transplant. Figure 1 (study flow diagram) provides details of the numbers of patients at each step in this recruitment process.<br><br>A small number of patients during the study interval were transplanted very quickly after activation on the waiting list (for example, those with significant accumulations of wait list points due to recently moving to our centre, or due to pre-existing diagnoses requiring treatment before activation). Figure 1 shows that 8/134 patients called in for transplant were not eligible due to not having had the necessary pre-consent consultation (i.e. were transplanted before the consultation was arranged). This is a potential source of bias, however the rate of this occurrence was very low. A small number of patients opted out of further contact about the trial whilst on the waiting list (34/405), or declined involvement following approach (2/50) - again see Figure 1. These are also potential sources of self-selection or bias, however the rates are again very low. Overall, the potential for recruitment bias is sufficiently low to be unlikely to have a relevant impact on the results.
Ethics oversight	Greater Manchester South national Research Ethics Committee (REC)<br>The Medicines and Healthcare Products Regulatory Agency (MHRA)<br>The National Health Service Research Authority (HRA)

Note that full information on the approval of the study protocol must also be provided in the manuscript.

Field-specific reporting

Please select the one below that is the best fit for your research. If you are not sure, read the appropriate sections before making your selection.

☒ Life sciences ☐ Behavioural & social sciences ☐ Ecological, evolutionary & environmental sciences

For a reference copy of the document with all sections, see nature.com/documents/nr-reporting-summary-flat.pdf

Life sciences study design

All studies must disclose on these points even when the disclosure is negative.

Sample size	No sample size calculation was performed as this was a single arm, phase 1 study with safety and feasibility as the primary considerations. A basic group size of 12 was selected, in accordance with the publication references in the Methods section of the manuscript (Julious et al.)
Data exclusions	There were no data exclusions.
Replication	There were no attempts at replication due to the study design (first in human, phase 1 trial).
Randomization	The allocation to groups was not random. Relevant covariates were controlled by matching using a pre-specified matching algorithm and a pool of historical controls, as described in the protocol and Methods section of the manuscript, and as reported in detail in the Results.
Blinding	Blinding was not relevant to this study design (phase 1 single contemporaneous arm).

Reporting for specific materials, systems and methods

We require information from authors about some types of materials, experimental systems and methods used in many studies. Here, indicate whether each material, system or method listed is relevant to your study. If you are not sure if a list item applies to your research, read the appropriate section before selecting a response.

Materials & experimental systems

n/a	Involved in the study	
☐	☒	Antibodies
☒	☐	Eukaryotic cell lines
☒	☐	Palaeontology and archaeology
☒	☐	Animals and other organisms
☐	☒	Clinical data
☒	☐	Dual use research of concern
☒	☐	Plants

Methods

n/a	Involved in the study	
☒	☐	ChIP-seq
☒	☐	Flow cytometry
☒	☐	MRI-based neuroimaging

Antibodies

Antibodies used	Antibodies were used as components of the following pre-packaged ELISA kits, used to generate results contained in the biomarker sections of this manuscript:<br><br>NGAL: Manufacturer: R&D Systems Inc. Catalog number: DLCN20.<br>KIM-1: Manufacturer: R&D Systems Inc. Catalog number: SKM100.<br>IL-18: Manufacturer: R&D Systems Inc. Catalog number: DL180.<br>GST-Pi: Manufacturer: Abcam. Catalog number: ab277423.<br>L-FABP: Manufacturer: Hycult Biotech. Catalog number: HK404.
Validation	All selected ELISA kits were validated by the manufacturer for use with serum and validation data for each kit is provided with the referenced kit manuals, available online. Normothermic perfusate differs from serum in that the protein compartment (prior to perfusion) consists of albumin only. Therefore, for each analyte a validation study was performed to optimise sample dilution, and ensure sufficient recovery. The procedure for doing this is provided in the Methods section of the manuscript. Pooled samples were assessed at at least three dilutions, and spikes of standard protein were used to calculate percentage recovery. All samples were analysed in duplicate and quality controlled; results where there was >30% difference between duplicates were rejected.

Clinical data

Policy information about clinical studies

All manuscripts should comply with the ICMJE guidelines for publication of clinical research and a completed CONSORT checklist must be included with all submissions.

Clinical trial registration	ISRCTN13292277
Study protocol	https://www.isrctn.com/editorial/retrieveFile/469583fd-c1e7-4208-8d6b-2544b882f82f/39309
Data collection	Data collection was performed at the single study site (Oxford Transplant Centre, Oxford, UK). The time period of recruitment was December 2021 - December 2022. The follow-up period (inclusive of 12-month follow-up for all participants) was December 2022 - December 2023.
Outcomes	The primary and secondary endpoints were specified in the study protocol (reproduced below), which was uploaded to the ISRCTN registry on-line in advance of the study opening. Outcomes were assessed at 30 days, 3 months, and 12 months post-transplant.<br><br>Primary outcome: 30-day graft survival (defined as a functioning graft and independence from dialysis).<br><br>Secondary outcomes:<br>3- and 12-month graft survival (defined as a functioning graft and independence from dialysis)<br>30-day, 3- and 12-month patient survival<br>Incidence and frequency of the use of dialysis in the first 7 days post-transplant<br>Incidence of functional Delayed Graft Function (fDGF), defined as the failure of serum creatinine to fall by at least 10% per day for the first 3 days.<br>Day 2 creatinine reduction ratio ((serum creatinine day 1 - serum creatinine day 2) / serum creatinine day 1)<br>Total proteinuria each day measured as milligrams of urinary protein by 24-hour urine collection, post-operative days 1-4<br>Total urine production, post-operative days 1-4<br>Incidence of Primary Non-Function (PNF), defined as persistent dialysis dependence at 3 months post-transplant<br>Estimated GFR (CKD-EPI formula)<br>Month 3 to month 12 serum creatinine gradient, defined as month 12 serum creatinine concentratino (umol/L) - month 3 serum creatinine concentration (umol/L).

Plants

Seed stocks	*Report on the source of all seed stocks or other plant material used. If applicable, state the seed stock centre and catalogue number. If plant specimens were collected from the field, describe the collection location, date and sampling procedures.*
Novel plant genotypes	*Describe the methods by which all novel plant genotypes were produced. This includes those generated by transgenic approaches, gene editing, chemical/radiation-based mutagenesis and hybridization. For transgenic lines, describe the transformation method, the number of independent lines analyzed and the generation upon which experiments were performed. For gene-edited lines, describe the editor used, the endogenous sequence targeted for editing, the targeting guide RNA sequence (if applicable) and how the editor was applied.*
Authentication	*Describe any authentication procedures for each seed stock used or novel genotype generated. Describe any experiments used to assess the effect of a mutation and, where applicable, how potential secondary effects (e.g. second site T-DNA insertions, mosiacism, off-target gene editing) were examined.*
